# Cancer susceptibility syndromes in children in the area of broad clinical use of massive parallel sequencing

**DOI:** 10.1007/s00431-015-2565-x

**Published:** 2015-05-16

**Authors:** Michaela Kuhlen, Arndt Borkhardt

**Affiliations:** Department of Pediatric Oncology, Hematology and Clinical Immunology, University Children’s Hospital, Medical Faculty, Heinrich-Heine-University, Moorenstr. 5, 40225 Duesseldorf, Germany

**Keywords:** Cancer susceptibility syndrome, Hereditary, Childhood, Next-generation sequencing, Chromothripsis, Mutation rate

## Abstract

Children diagnosed with cancer are considered for inherited cancer susceptibility testing according to well-established clinical criteria. With increasing efforts to personalize cancer medicine, comprehensive genome analyses will find its way into daily clinical routine in pediatric oncology. Whole genome and exome sequencing unavoidably generates incidental findings. The somatic “molecular make-up” of a tumor genome may suggest a germline mutation in a cancer susceptibility syndrome. At least two mechanisms are well-known, (a) chromothripsis (Li-Fraumeni syndrome) and (b) a high total number of mutational events which exceeds that of other samples of the same tumor type (defective DNA mismatch repair). Hence, pediatricians are faced with the fact that genetic events within the tumor genome itself can point toward underlying germline cancer susceptibility. Whenever genetic testing including next-generation sequencing (NGS) is initiated, the pediatrician has to inform about the benefits, risks, and alternatives, discuss the possibility of incidental findings and its disclosure, and to obtain informed consent prior to testing.

*Conclusions*: Genetic testing and translational research in pediatric oncology can incidentally uncover an underlying cancer susceptibility syndrome with implications for the entire family. Pediatricians should therefore increase their awareness of chances and risks that accompany the increasingly wide clinical implementation of NGS platforms.

**Table:** 

**What is Known**: • *The proportion of cancers in children attributable to an underlying genetic syndrome or inherited susceptibility is unclear*. • *Pediatricians consider children diagnosed with cancer for inherited cancer susceptibility according to well-established clinical criteria*.
**What is New**: • *Genetic testing of tumor samples can incidentally uncover an underlying cancer susceptibility syndrome*. • *Findings in tumor genetics can be indicative that the tumor arose on the basis of the child*’*s germline alteration*, (*a*) *chromothripsis and* (*b*) *a high total number of mutational events which exceeds that of other samples of the same tumor type*.

## Introduction

Until now, the proportion of cancers in children and adolescents attributable to an underlying genetic syndrome or inherited susceptibility is unclear. In the early 1990s, the inherited fraction of childhood cancer was estimated at 1–10 % [29]. A recent report from the Pediatric Cancer Genome Project/St. Jude Children’s Research Hospital determined an incidence of 16.0 % in patients with solid tumors, 8.6 % with brain tumors, and 3.9 % with leukemias. The report initially focused on 23 well-known cancer predisposition genes and 8 genes that predispose to pediatric cancer with a high penetrance [47]. The most frequently affected genes included TP53, APC, and BRCA2. Additional analyses were expanded to 565 genes that are known to play a role in various steps and pathways of cellular transformation. Identified variants were classified as pathologic, likely pathologic, uncertain significance, likely benign, and benign. Taking the larger gene-set into account, the overall prevalence of an inherited mutation increased only slightly, with a pathologic or likely pathologic variant being detected in 8.6 % of all patients and 4.6 % of patients with leukemia. However, the spectrum of tumors sequenced was not numerically representative of the spectrum of childhood tumors, and the mutation frequencies may be skewed accordingly. In a hereditary cancer risk assessment study in survivors of childhood cancer, a genetic counselor considered 29 % of the survivors as eligible for further genetics evaluation [19].

However, in the era of high-throughput sequencing in which new cancer susceptibility syndromes (CSS) and mechanisms are increasingly discovered—did we so far maybe just see the tip of the iceberg?

### Current clinical approach to CSS

Pediatric oncologists consider children diagnosed with cancer and their families for inherited cancer susceptibility according to well-established criteria [20]. These comprise patient-specific constellations including (i) rare tumors commonly associated with cancer predisposition (e.g., adrenocortical carcinoma), (ii) bilateral or multifocal tumors (e.g., Wilms’ tumor), (iii) cancer diagnosis at a younger than expected age (e.g., thyroid carcinoma), (iv) multiple synchronous or metachronous tumors, (v) additional conditions (e.g., axillary freckling) indicative of an underlying syndrome, and (vi) suspicious family features. These might include (a) familial clustering of the same or closely related cancers, (b) cancer diagnoses in two or more first-degree relatives, (c) tumor patterns associated with a specific cancer predisposition syndrome, (d) exceptional young age at diagnosis, (e) sibling with childhood cancer, and (f) consanguineous parents.

Li-Fraumeni syndrome (LFS) is one of the most striking familial cancer predisposition syndromes. It is clinically and genetically heterogeneous and characterized by autosomal dominant inheritance and early onset of tumors, multiple tumors within one individual, and multiple affected family members. LFS presents with a variety of tumor types with soft tissue sarcomas, osteosarcomas, breast cancer, brain tumors, leukemia, and adrenocortical carcinoma being the most common tumor types. Comprehensive surveillance protocols have been implemented and proven efficiency in terms of superior survival [46]. Table 1 lists common hereditary cancer susceptibility syndromes sorted by the underlying mechanism. The American College of Medical Genetics and Genomics and the National Society of Genetic Counselors just published the latest referral indications for cancer predisposition assessment [13]. However, due to de novo mutations, incomplete penetrance of inherited mutations, and variable phenotype/genotype correlations, the family history may not in all cases be helpful. For example, up to 25 % de novo events of TP53 mutations are reported in Li-Fraumeni syndrome [6]. In most other cases of CSS, however, the proportion of inherited susceptibility versus de novo mutations remains unknown.

**Table 1:** Examples of common hereditary cancer predisposition syndromes

Syndrome	Gene(s)	Inheritance	Clinical characteristics	Tumor types	Cancer risk
DNA damage repair defects/genetic instability					
Ataxia telangiectasia (AT) *	ATM	AR	Progressive ataxia, central nervous system degeneration, growth deficiency, ocular and facial telangiectasias, immunodeficiency, infertility, premature aging	Leukemia, lymphoma, carcinoma	10–38 % overall cancer risk 70-fold increased leukemia risk (T-ALL, T-PLL) 250-fold increased lymphoma risk (B cell)
Bloom syndrome (BS)	BLM	AR	Short stature, immunodeficiency, malar rash, microcephaly, high-pitched voice, hypogonadism	Leukemia, lymphoma	50 % overall cancer risk 15 % leukemia risk 15 % lymphoma risk
Constitutional mismatch repair-deficiency syndrome (CMMR-D)	MLH1, MSH2, MSH6, PMS2	AR	Multiple café au lait (CAL) spots, features reminiscent of NF1	Pediatric brain tumors, colorectal cancers, ALL, AML, lymphoma, early onset gastrointestinal or gynecological cancers	Biallelic mutations at very high risk
Fanconi anemia (FA)	FANCA, C, D1, D2, E, F, G, I, J, L, M, RAD51C, SLX4/BTBD12 FANCB	AR X-linked	Bone marrow failure, growth failure, radial ray abnormalities, renal abnormalities, CAL spots, hypopigmentation, congenital heart disease, microphthalmia, ear anomalies/deafness, hypogonadism; up to 25 % phenotypically normal	Leukemia (MDS, AML), squamous cell carcinoma, gynecological tumors, brain tumors, Wilms tumor, neuroblastoma	25 % cumulative risk of hematologic malignancy by age 45 7 % MDS 9 % (500-fold increased risk of) AML
Li-Fraumeni syndrome (LFS)	TP53	AD up to 25 % de novo mutations	beyond classical LFS malignancies phenotypically normal	Soft tissue sarcoma, osteosarcoma, breast cancer, adrenocortical carcinoma (ACC), leukemia, brain tumors (glioblastoma multiforme, high-grade astrocytoma/primitive neuroectodermal tumor, medulloblastoma, choroid plexus carcinoma)	90 % lifetime risk to develop cancer 1–3 % ALL (hypodiploid ALL)
Nijmegen breakage syndrome (NBS)	NBS1	AR	Microcephaly, prominent midface, receding mandible, CAL, recurrent infections, bone marrow failure	NHL, DLBCL, Burkitt lymphoma, T-LBL/-ALL, AML, Hodgkin lymphoma, medulloblastoma, rhabomyosarcoma	40 % cancer risk by the age of 20 years
Bone marrow failure (BMF) syndromes: ribosome biogenesis and/or telomere maintenance anomalies					
Congenital amegakaryocytic thrombocytopenia (CAMT) type I / II	MPL	AR	Thrombocytopenia and megakaryocytopenia with no physical anomalies	MDS/AML	Unknown
Diamond blackfan anemia (DBA)	RPS19, RPS24, RPS17, RPL35A, RPL5, RPL11, RPS7, RPS26, RPS10, GATA1	AD Majority sporadic	Normochromic macrocytic anemia, reticulocytopenia, and nearly absent erythroid progenitors in the bone marrow, growth retardation, craniofacial, upper limb, heart, and urinary system congenital malformations, persistence of hemoglobin F	Adenocarcinoma of the colon, sarcoma, genital cancer, MDS/AML, ALL	5.4 %-fold increased cancer risk
Dyskeratosis congenital (DC)	DKC1, CTC1, TERC, TERT, TINF2, NOP10, NHP2, WRAP53	X-linked	Triad of abnormal skin pigmentation, nail dystrophy, and leukoplakia of the oral mucosa	MDS/AML	3–33 % leukemia risk
Shwachman-Diamond syndrome (SDS)	SBDS	AR (considered)	Exocrine pancreatic insufficiency, hematologic abnormalities (pancytopenia), skeletal abnormalities	MDS/AML, ALL	5–24 % leukemia risk
Severe congenital neutropenia (SCN) (Kostmann syndrome) *	ELANE, HAX1	AD AR	Congenital neutropenia, recurrent/persistent infections, omphalitis	MDS/AML	8–25 % leukemia risk
Thrombocytopenia and absent radii syndrome (TAR)	RBM8A and/or microdeletion 1q21.1	Unclear	Reduction in the number of platelets and absence of the radius	MDS/AML	Unknown
Cell cycle/differentiation defects (RAS pathway dysfunction)					
CBL syndrome	CBL	AD	Dysmorphic facial features, short neck, developmental delay, hyperextensible joints, and thorax abnormalities with widely spaced nipples	JMML	Unknown
Neurofibromatosis type I (NF1)	NF1, SPRED1	AD	CAL, axillary/inguinal frecking, Lisch nodules, bony dysplasia, seizures, learning difficulties, sphenoid wing abnormalities	CMML/JMML, AML, neurofibroma, optic pathway glioma, peripheral nerve sheath tumor, astrocytoma, paraganglioma/pheochromocytoma	200–500-fold increased JMML risk 11 % MDS 5-fold increased brain tumor risk almost 100 % neurofibroma risk
Noonan/Noonan-like syndrome	PTPN11, HRAS, KRAS, NRAS, RAF1, SOS1, BRAF, SHOC2, MEPK1	AD	Short stature, short webbed neck, lymphedema, hypertelorism, coarse facies, CAL, pulmonary valve stenosis, pectus excavatum, wide and low-set nipples, cardiomyopathy, bleeding disorders	Self-resolving myeloproliferative disease (MPD/TMD) and JMML, CMML, ALL, neuroblastoma, rhabdomyosarcoma	MPD/JMML in pts with PTPN11
Transcription factors/pure familial leukemia syndromes					
Familial CEBPA leukemia	CEBPA	AD	None	MDS/AML	FAB M1/M2 highly penetrant
Familial ETV6 / ALL syndrome	ETV6	AD	Thrombocytopenia	MDS/AML, MPAL, ALL, multiple myeloma, colon cancer	Unknown
Familial platelet disorder with predisposition to myeloid malignancy (FPD/AML)	RUNX1 (dominant)	AD	Mild to moderate thrombocytopenia, platelet function abnormalities	MDS/AML	35 % AML risk
Familial PAX5 syndrome	PAX5	AD	None	ALL	30 % penetrance in PAX5 SNP allele carriers PAX5 c.547G > A
MonoMac	GATA2	AD	Monocytopenia, NK cell lymphopenia, infections	MDS/AML	50 % leukemia risk
Immunodeficiencies					
Wiskott-Aldrich syndrome (WAS)	WAS	X-linked	Eczema, thrombocytopenia, immunodeficiency	Diffuse large B cell lymphomas, non-Hodgkin’s lymphoma of larynx, leukemia, cerebellar astrocytoma, Kaposi sarcoma, smooth muscle tumors	5–13 % lymphoid malignancies
X-linked lymphoproliferative (XLP) syndrome type I / II	SH2D1A XIAP, SAP	X-linked	Severe immune dysregulation often after viral infection, typically with Epstein-Barr virus (EBV), severe or fatal mononucleosis, acquired hypogammaglobulinema, (HLH), lymphomatoid granulomatosis	Hemophagocytic lymphohistiocytosis (HLH), non-Hodgkin lymphoma	Unknown
Autoimmune lymphoproliferative syndrome (ALPS) type IA/B/II	CD95 CD95L CASP10 IL12RB1	AD AR in ALPS1A	Lymphadenopathy with hepatosplenomegaly and autoimmune cytopenias, hypergammaglobulinema	Hodgkin (HL) and non-Hodgkin (NHL) lymphoma, carcinoma (thyroid, breast, skin, tongue, liver), multiple neoplastic lesions (thyroid/breast adenomas, gliomas)	14-fold NHL risk 51-fold HL risk
IL2-inducible T cell kinase deficiency	ITK	AR	Fever, lymphadenopathy, splenomegaly, EBV associated lymphoproliferation	Hodgkin lymphoma,	Unknown
Unknown					
Familial mosaic monosomy 7	Unknown	Unknown	Early-childhood onset of bone marrow insufficiency / failure	MDS, AML	Very high, fatal outcome
Congenital syndromes/aneuploidy					
Beckwith-Wiedemann syndrome (BWS)	p57, H19, LIT1, ICR1, CDKN1C, NSD1	complex (AD, genomic imprinting, pUPD)	Overgrowth syndrome, marcoglossia, omphalocele, hemihypertrophy, neonatal hypoglycemia	Wilms tumor, hepatoblastoma, adrenal carcinoma, rhabdomyosarcoma	8.6 % cancer risk, depending on subtypes highest risk in patients with hemihypertrophy and organomegaly
Cowden syndrome type I-VI (CWS)	PTEN, SDHB, SDHD, KLLN	AD	Hamartomatous polyps of the gastrointestinal tract, mucocutaneous lesions, cobblestone-like papules of the gingiva and buccal mucosa, multiple facial trichilemmomas	Dysplastic gangliocytoma of the cerebellum (Lhermitte-Duclos), colon, breast and thyroid cancer	Lifetime risk 25–30 % breast cancer 10 % thyroid cancer 5–10 % endometrial/uterine cancer
Denys-Drash syndrome (DDS)	WT1 (dominant)	Usually sporadic	Diffuse mesangial sclerosis leading to early endstage renal disease, disorder of sexual development in XY patients	Wilms tumor, gonadoblastoma	Almost 100 % Wilms tumor
Down syndrome (DS)	Trisomy 21	n.a.	Facial dysmorphism, mental retardation, hypotonia, congenital heart disease	TMD, AML, ALL	10 % TMD, 1–2 % ALL/AML 10–20-fold increased leukemia risk 500-fold increased risk of AMKL
Familial Adenomatous Polyposis (FAP) syndrome	APC	AD	Intestinal polyposis, osteomas, fibromas, sebaceous cysts, dental abnormalities	Colon, thyroid, stomach, and intestinal cancer, hepatoblastoma, desmoid tumors, medulloblastoma	Almost 100 % colorectal cancer
Familial neuroblastoma	ALK, PHOX2B	AD	None	Neuroblastoma	Unknown
Familial Pleuropulmonary blastoma tumor predisposition syndrome	DICER1	AD	Pulmonary cysts, multinodular goiter	PPB, cystic nephroma, Sertoli-Leydig cell tumors, rhabdomyosarcoma, supratentorial primitive neuroectodermal tumor, intraocular medulloepithelioma	Variable penetrance, exact rest unknown
Hereditary paragangliomas and pheochromocytoma syndrome (HPPS)	SDHB	AD	None	Paraganglioma, pheochromocytoma, renal, thyroid	>70 % with metastatic disease 12 % GISTs
Multiple endocrine naeoplasia type I (MEN1)	MEN1	AD	None	Pancreatic islet cell tumor, pituitary adenoma, parathyroid adenoma	10 % carcinoid tumors
Multiple endocrine neoplasia type II (MEN2A, MEN2B)	RET	AD	Mucosal neuroma (intestinal tract, tongue, lips), marfanoid habitus	Medullary thyroid carcinoma, pheochromocytoma, parathyroid hyperplasia	100 % risk of developing medullary thyroid carcinoma in MEN2A
Nevoid basal cell carcinoma syndrome (NBCCS) / Gorlin syndrome	PTCH1, 2, SUFU	AD	Macrocephaly, hypertelorism, palmar or plantar pits, rib abnormalities, ectopic calcification of the falx cerebri	Basal cell carcinoma, desmoplastic medulloblastoma, ovarian fibromas	90 % basal-cell carcinoma, 5 % medulloblastoma
Peutz Jeghers syndrome (PJS)	STK11	AD	Melanocytic macules of the lips, buccal mucosa, digits, multiple gastrointestinal hamartomatous polyps	Intestinal, ovarian, pancreatic, breast cancers	55 % gastrointestinal cancer 45 % breast cancer
Familial retinoblastoma syndrome (RB)	RB1	AD	Leukocoria	Retinoblastoma, osteosarcoma, melanoma, glioma, carcinoma	80 % bilateral retinoblastoma 20 % unilateral retinoblastoma
Rhabdoid tumor predisposition syndrome	SMARCB1/INI1	Unclear, up to 21 % de novo mutations	None	Rhabdoid tumor, medulloblastoma, choroidplexus tumor, schwannoma	Penetrance unclear
Rubinstein-Taybi syndrome (RSTS)	CREBBP	AD	Short stature, learning difficulties, distinctive facial features, broad thumbs and first toes, microcephaly, growth retardation	Neuroblastoma, medulloblastoma, oligodendroglioma, meningeoma, pheochromocytoma, rhabdomyosarcoma, leiomyosarcoma, leukemia, lymphoma	Unknown
Tuberous sclerosis complex (TSC)	TSC1/TSC2	AD	Tubers, heterotopia, central nervous system migrational/psychomotor delay, seizures, renal/bone cysts	Subependymal giant cell astrocytoma, hamartoma, renal angiomyolipoma, renal cell carcinoma, cardial rhabdomyoma, renal angiomyolipoma	4 % renal cell carcinoma 14 % giant cell astrocytoma
Lynch syndrome type I / II	MLH1, MSH2, MSH6, PSM2	AD	CAL	Colorectal cancer, glioblastoma multiforme, medulloblastoma	Depending on subtype 50.4 % cumulative risk for colorectal cancer at the age of 70
WAGR syndrome	WT1	AD	Aniridia, genitourinary abnormalities, mental retardation	Wilms tumor, gonadoblastoma	High percentage of bilateral Wilms tumors

*And cell cycle regulation

### Personalized medicine

With the ongoing efforts to personalize cancer medicine, comprehensive genome analyses will increasingly find its way into daily clinical routine in pediatric oncology. In the recently established German INdividualized therapy FOr Relapsed Malignancies in childhood (INFORM) project, this idea has been introduced for pediatric patients with relapsed or refractory high-risk disease without further standard of care therapy options. Individual tumor samples are characterized on the molecular level by next-generation sequencing (NGS) to establish a “fingerprint” of the tumor to identify promising targets for a successful relapse therapy [10].

Other such examples in which the detection of specific mutations has already led to a change of therapy of course also exist. Recently, a new leukemia subtype of high-risk B-precursor acute lymphoblastic leukemia (ALL), called Ph-like ALL, was characterized. Besides its Ph- or BCR-ABL-like transcriptional profile, no translocation t(9;22) or BCR/ABL rearrangement, respectively, is present. Instead, multiple other genetic alterations can be detected, which are potentially druggable by tyrosine kinase inhibitors or other targeted therapies [18, 24, 36, 37]. In pediatric low-grade astrocytoma, the BRAF V600E-mutation was identified as a frequent genomic aberration activating the MAPK pathway. Tumors carrying this mutation show significantly increased BRAF and CCND1 levels [33]. Since its discovery, the BRAF V600E-mutation has been described in an increasing number of pediatric central nervous system (CNS) tumors [8, 11, 40, 41]. Targeted therapies such as the BRAF inhibitor vemurafenib and MEK1/2 inhibitors are available and some encouraging examples of effective therapies even in very aggressive tumor types have already been reported, such as the successful treatment of a 12-year-old child with relapsed glioblastoma multiforme with vemurafenib [38]. With the identification of a highly recurrent genetic alteration and its resulting fusion protein in ependymoma, the C11orf95-RELA protein, a further potentially druggable target was identified and specific therapy will hopefully be available in the near future [31]. We might also hypothesize that children with hereditary cancer syndromes like the so-called rasopathies might soon benefit from targeted therapy, as the underlying genetic alterations are highly recurrent [1, 9].

### Next-generation sequencing

Due to rapid technical advances in the field of NGS, tumor (including leukemia) genomes can nowadays comprehensively be analyzed within few days. Today’s state of the art in high-throughput sequencing already allows the usage of whole genome sequencing for research projects and of whole exome sequencing for daily clinical routine. However, the likelihood of identifying contemplable mutations is highly dependent on the relative ability of the sequencing approach to find these mutations. Computational processing, analyzing, and interpreting the massive amounts of data and genetic variants produced by NGS still remains challenging and requires comparisons with databases such as dbSNP and 1000 genomes project [3, 16]. Another valuable resource in interpreting own experimental data is the ExAC browser provided by the Broad Institute at www.exac.broadinstitute.org. It meanwhile provides exome data from more than 60,000 unrelated individuals. Before definitive conclusions can be drawn, the functional consequences of identified mutations on protein structure and function often have to be demonstrated experimentally [43]. In addition, a frequent conceptual misunderstanding relates to the fact that even a mutation with profound impact on protein function does not automatically proves its pathogenicity and disease-causing effect.

Each of us carries an average of approximately 3000 single nucleotide polymorphisms (SNPs) in terms of individual SNPs. To generate a personal cancer genome signature for molecular targeted therapy, it is important to discriminate between these individual SNPs and somatic (tumor) mutations. Thus, comparing the NGS data of tumor versus germline DNA is a condition sine qua non to identify the somatically acquired genetic variants of the tumor.

However, NGS not only generates focused genetic results with precise clinical implications for treatment but also so-called incidental findings with possible, limited, or unknown clinical impact or might even uncover an underlying susceptibility to cancer and other hereditary diseases. Such incidental findings are divided into “anticipatable” and “unanticipatable” ones. The former is a finding that is known to be associated with the test and is possible to be found. The latter could not have been anticipated given the current state of scientific knowledge [17]. Hence, treating physicians will increasingly be faced with such incidental genetic findings and the difficulties of interpreting and reporting these results.

Moreover, the pediatric oncologist is confronted with one new situation in particular: the fact that genetic events within the tumor genome itself can point toward underlying germline cancer susceptibility. Thus, even if not initially aimed to detect a CSS, the somatic “molecular make-up” of the tumor genome may suggest a germline mutation in a CSS gene.

Up to now, there are two well-known findings in tumor genetics which can be indicative that the tumor arose on the basis of the child’s germline alteration, (a) chromothripsis and (b) a high total number of mutational events which exceeds that of other samples of the same tumor type.

- The phenomenon of *chromothripsis* was first reported by Stephens in 2011 [44]. The term “chromothripsis” (“chromo” from chromosome; “thripsis” for shattering into pieces) describes the shattering of a chromosome or a chromosomal region into tens to hundreds of pieces and locally clustered reassembling of some of the genomic fragments while others are lost to the cell.

According to Stephens [44], chromothripsis is defined by six features: (1) rearrangements localized within the genome, (2) oscillating changes of the copy number profile between one and two copies, whereby (3) loss of heterozygosity (LOH) causes a copy number of one, and retaining heterozygosity a copy number of two, (4) clustering of breakpoints across the chromosome, (5) conjunction of two remote chromosome fragments, and (6) joining rearrangements between two chromosome arms with clustering at the breakpoints. Rapid oscillations between copy number states one and two within the whole or parts of the chromosome characterizes the copy number profile in case of chromothripsis.

In contrast to common theories of cancer evolution through progressive accumulation of genomic alterations such as oncogene activation and tumor suppressor loss through environmental and lifestyle factors in adults, chromothripsis as a single catastrophic event might be involved in the development of a variety of cancers in childhood. It can cause the formation of new gene fusions, disruption of tumor suppressors, and amplification of oncogenes [35, 44]. In adults, 2–3 % of all cancers show evidence of chromothripsis; in bone cancers, this incidence is especially high with 25 % [44]. The impact of chromothripsis on cancer gene function and cancer development in childhood has already been demonstrated for many different tumor entities, e.g., ALL, AML, ependymoma, medulloblastoma, neuroblastoma, and retinoblastoma [4, 23, 26, 28, 30, 31, 35]. In addition, chromothripsis has been associated with poor prognosis in neuroblastoma [28]. A list of pediatric tumors, in which chromothripsis has been described, is given in Table 2. Conversely, alterations in TP53 have been shown for low-hypodiploid ALL but without chromothriptic pattern [15].

**Table 2:** Examples of (pediatric) tumors associated with chromothripsis

Tumor	References
Burkitt lymphoma *	Sarova et al., Cancer Genet 2014
Brain tumors • Ependymoma • High-grade gliomas • Medulloblastoma -Sonic-Hedgehog -Group 3	• Parker et al., Nature 2014 • Zhao et al., Neuro Oncol 2014 -Rausch et al., Cell 2012 -Northcott et al., Nature 2012
Hodgkin lymphoma *	Nagel et al., Genes Chromosomes Cancer 2013
Leukemia • AML • ALL (iAMP21)	• Rausch et al., Cell 2012 • Harrison et al., Blood 2015; Li et al., Nature 2014
Neuroblastoma	Ambros et al., Frontiers in Oncology 2014; Boeva et al., PLoS One 2013; Molenaar et al., Nature 2012
Osteosarcoma *	Stephens et al., Cell 2011
Phaeochromocytoma (PCC) / Paraganglioma (PGL) *	Flynn et al., J Pathol 2014
Retinoblastoma	McEvoy et al., Oncotarget 2014

*Described in adult tumor samples

(b) To provide a comprehensive landscape of somatic genomic alterations (termed mutational signatures) in cancer genomes, numerous cancers have been profiled by DNA sequencing [2, 34, 45]. The occurring genomic alterations are presumably caused by defective DNA replication or repair and exogenous or endogenous mutagen exposure and include substitutions, insertions or deletions, rearrangements, copy number alterations, completely new sequences from exogenous sources, and combinations of all these possibilities. The prevalence of such mutations is highly variable between cancer (sub)types [2, 22]. Due to extensive exposure to carcinogens, small cell lung cancer (tobacco) and malignant melanoma (ultraviolet light) show the highest somatic mutation prevalence (over 100/Megabase (Mb)). In contrast, the mutation rate in pediatric cancers is lowest (0.1/Mb; approximately one change across the entire exome) as chronic mutagenic exposure plays a minor part in carcinogenesis in childhood [22]. An outline of mutation frequencies in various (pediatric) cancer types is given in Table 3.

**Table 3:** Examples of mutation frequencies in (pediatric) tumors

Malignancy	Mutations (range)	Reference
AML^a^	0.37 per Mb (0.01–10) of coding sequence	Lawrence et al., Nature 2013
Ependymoma, intracranial^b^	12.8 ± 10.6 mutations (range 5 to 34) per tumor	Bettegowda et al., Oncotarget 2013
Ependymoma, spinal cord^b^	12.9 ± 6.4 mutations (range 2 to 23) per tumor	Bettegowda et al., Oncotarget 2013
Ewing^a^	0.15 per Mb of coding sequence	Brohl et al., PLoS Genet 2014
Glioblastoma multiforme^b^	1.4 per Mb	Cancer Genome Atlas Research Network, Nature 2008
Glioblastoma, non-brainstem pediatric	23.5 ± 11.2 mutations (range 4–46) per tumor	Bettegowda et al., Oncotarget 2013
MDS^b^	3 (0–12) mutations per sample in 104 cancer genes	Haferlach et al., Leukemia 2014
Medulloblastoma	8.3 non-synonymous SNVs per sample 0.35 non-silent mutations per megabase	Parsons et al., Science 2011 Pugh et al., Nature 2012
Neuroblastoma	0.60 per Mb of coding regions	Pugh et al., Nature Genet 2013
Rhabdoid cancers	0.19 per Mb (0–0.45) of coding regions	Lee et al., J Clin Invest 2012
Xanthoastrocytoma, pleomorphic^b^	9.5 ± 8.5 mutations (range 1 to 28) per tumor	Bettegowda et al., Oncotarget 2013

^a^Tumor samples not specified

^b^Described in adult tumor samples

Alexandrov et al. [2] described a mutational signature with very large numbers of substitutions and small indels, the latter at short nucleotide repeats and with overlapping microhomology at breakpoint junctions, termed “microsatellite instability,” which is characteristic of cancers with defective DNA mismatch repair and may suggest constitutional mismatch repair-deficiency syndrome (CMMR-D) in childhood.

As was shown by Rausch et al. [35], the single nucleotide variant (SNV) rate of children with Sonic-Hedgehog medulloblastoma (SHH-MB) is clearly higher (24 tumor-specific SNVs) in the case of inherited TP53 mutations compared to sporadic pediatric medulloblastoma samples (average 5.7 non-synonymous SNVs per sample; [32]). Thus, comparing the patient’s SNV with the average SNV rate of a given tumor entity, an increased mutation frequency (SNV rate) detected by NGS of the tumor again may point to an underlying CSS (Li-Fraumeni syndrome).

### Ethical and legal issues

“Are our other children at an increased risk of developing cancer?” Parents of a child diagnosed with cancer frequently raise this question. Up to now, pediatric oncologists mostly reassure them that cancer in children usually is not hereditary but an exceptionally bad stroke of fate. However, will this statement still hold true in the future with ever-increasing knowledge about underlying cancer predisposition syndromes and inherited cancer susceptibilities in childhood?

The incidental finding of chromothripsis and its association with Li-Fraumeni syndrome in SHH-MB patients very well demonstrates the far-reaching consequences of translational research and genetic testing in pediatric oncology with its challenges for scientists, treating physicians, and the affected child and his entire family.

By detecting chromothripsis in a tumor, further genetic testing for germline p53 mutations is highly advisable as this phenomenon might be attributable to an underlying Li-Fraumeni syndrome. The latter obviously represents an important piece of clinical information as it will guide treatment, surveillance, and further early cancer screening programs [21, 46].

According to the recommendations of national and international human genetic societies and the legislation of most European countries, prior to genetic testing, the child (wherever possible) and the parents must be informed in detail, preferences as to which findings should be reported must be assessed, and written informed consent must be obtained. This is a well-established standard of care for targeted molecular testing an affected individual or suspected carrier for a specific hereditary condition. However, NGS is likely, apart from the initial indication to perform it, to uncover incidental findings, such as an underlying CSS as well as non-cancer-related germline mutations (e.g., CFTR, Huntington’s disease) with varying clinical importance for the patient. In order to comply with the aforementioned recommendations, this would require extensive genetic counseling of the child/parents of a child diagnosed with cancer undergoing NGS of the tumor prior to testing, which would have to encompass both incidental findings with possible, limited, or unknown clinical impact and numerous results unrelated to the indication for NGS [42]. We believe that this is highly impractical in the daily life of a pediatric hemato-oncologist as disclosing the diagnosis of cancer itself is overwhelming and dramatically limits the child’s/parents’ receptivity, and NGS of the tumor often has to be initiated at the time of diagnosis. However, whenever NGS is initiated, the treating physician has an obligation to discuss the full range of generated data and the possibility of incidental findings and its disclosure with the child/parents. Furthermore, the ordering physician is responsible for obtaining informed consent and providing pre- and post-test counseling. Thus, regarding the child’s/parents’ autonomy and both their right to access all NGS data and their “right not to know,” they should be informed of the benefits, risks, and alternatives of genetic testing in detail [5, 7, 12]. When the patient/parent refuses to be informed about incidental findings, even if disclosure leads to beneficial interventions, the physician must ensure that adequate information has preceded this refusal. However, most clinicians do not have sufficient training in NGS and need to be extensively trained for clinical translation and reporting of NGS data.

In contrast to the standards for genetic testing in adults, predictive testing in pediatric patients is only recommended when the disease is associated with childhood onset and only with available effective screening and/or intervention options [7, 39]. Refraining from predictive testing of children allows them to autonomously make this decision once they reach adulthood.

Last but not least, identifying children with hereditary cancer predispositions has immediate consequences for the entire family (siblings, parents, and extended family) [20, 25, 42]. Due to the young age of the index patient, potentially affected relatives might as well be young and yet asymptomatic. Having been tested themselves might—depending on the outcome—influence their family planning but will of course also provide an excellent opportunity to initiate early cancer surveillance programs which they will benefit from. However, genetic testing and tumor surveillance can have deeply affecting psychological consequences for the child and the family, emotional support should thus be in place for the families.

Clear legislation on returning genetic results in oncology are still missing. Lolkema et al. have thoroughly addressed the accompanying ethical, legal, and counseling challenges [25]. Comprehensive ethical recommendations on how to report research results to patients and parents are, for example, given by the American College of Medical Genetics and Genomics, the Boston Children’s Hospital, the American Academy of Pediatrics, the “EURAT” (Ethical and legal aspects of whole human genome sequencing) project of the Marsilius Kolleg of Heidelberg University, and the Leopoldina National Academy of Sciences Germany [5, 12, 14, 27, 39]. However, their practical implementation in day-to-day clinical life remains challenging.

## Conclusions

Genetic testing and translational research in pediatric oncology provides new and exciting insights into the evolution and pathogenesis of childhood cancer. On the other hand, it can incidentally uncover an underlying cancer susceptibility syndrome with implications not only for the child but also for the entire family. Pediatric oncologists should therefore increase their awareness of chances and risks that accompany the increasingly wide clinical implementation of NGS platforms [42, 43].

## Abbreviations

ALL: Acute lymphoblastic leukemia
CMMR-D: Constitutional mismatch repair-deficiency
CSS: Cancer susceptibility syndromes
DGV: Database of genomic variants
INFORM: Individualized therapy for relapsed malignancies in childhood
LFS: Li-Fraumeni syndrome
LOH: Loss of heterozygosity
Mb: Megabase
NGS: Next-generation sequencing
SHH-MB: Sonic-Hedgehog medulloblastoma
SNPs: Single nucleotide polymorphisms
SNVs: Single nucleotide variants
